# Prediction of volume response under open-chest conditions during coronary artery bypass surgery

**DOI:** 10.1186/cc6181

**Published:** 2007-11-22

**Authors:** Michael Sander, Claudia D Spies, Katharina Berger, Herko Grubitzsch, Achim Foer, Michael Krämer, Matthias Carl, Christian von Heymann

**Affiliations:** 1 Department of Anesthesiology and Intensive Care Medicine, Charité Universitätsmedizin Berlin, Campus Virchow Klinikum and Campus Charité Mitte, Augustenburger Platz 1, 13353 Berlin, Germany; 2 Department of Cardiovascular Surgery, Charité Universitätsmedizin Berlin, Campus Virchow Klinikum and Campus Charité Mitte, Augustenburger Platz 1, 13353 Berlin, Germany

## Abstract

**Introduction:**

Adequate fluid loading is the first step of hemodynamic optimization in cardiac patients undergoing surgery. Neither a clinical approach alone nor conventional parameters like central venous pressure (CVP) and pulmonary capillary wedge pressure (PCWP) are thought to be sufficient for recognizing fluid deficiency or overload. The aim of this study was to evaluate the suitability of CVP, PCWP, global end-diastolic volume index (GEDVI), pulse pressure variation (PPV), and stroke volume variation (SVV) for predicting changes in the cardiac index (CI) and stroke volume index (SVI) after sternotomy.

**Methods:**

In 40 patients, CVP, PCWP, GEDVI, PPV, SVV, and the CI were measured at two points of time. One measurement was performed after inducing anesthesia and one after sternotomy.

**Results:**

A significant increase in heart rate, CI, and GEDVI was observed during the study period. CVP, SVV, and PPV decreased significantly. There were no significant correlations between CVP and PCWP and changes in CI. In contrast, GEDVI, SVV, and PPV significantly correlated with CI changes. Only relative changes of GEDVI, SVV, and PPV predicted changes in SVI.

**Conclusion:**

During cardiac surgery and especially after sternotomy, CVP and PCWP are not suitable for monitoring fluid status. Direct volume measurement like GEDVI and dynamic volume responsive measurements like SVV and PPV may be more suitable for monitoring the volume status of patients, particularly under open-chest conditions.

## Introduction

Adequate fluid loading is the first step in the hemodynamic optimization of surgical patients. This is especially true for cardiac patients, who, on the one hand, may have a fluid deficiency because of preoperative fasting but, in turn, may only restrictedly tolerate rapid fluid substitution depending on the underlying cardiac disease. An exact estimation of volume status is particularly difficult in these patients. The clinical approach alone is often not sufficient for the early recognition of fluid deficiency or overload to implement targeted treatment [1]. Thus, volume status imbalances frequently are not recognized at all or are recognized too late, which may have drastic consequences for the hemodynamic stability of these patients [1,2].

Thus far, filling pressures have been used most often in the clinical routine to assess the hemodynamic status and the volume status. In a nationwide survey of internal medicine and surgical intensive care units, central venous pressure (CVP) was named in 90% of the cases as the monitoring procedure of choice for volume therapy, followed by pulmonary capillary wedge pressure (PCWP) with almost 60% [3]. A more recent survey of cardiosurgical intensive care specialists showed that CVP is used for monitoring volume therapy 87% of the time, followed by mean arterial blood pressure with 84% and PCWP with 30% [4]. Parameters allowing a directly measured approximation of intrathoracic fluid loading have been used in the clinical routine for some years. These parameters may contribute to more targeted and optimized volume therapy. To estimate the volume status, the global end-diastolic volume index (GEDVI) might be of more use than filling pressures [5]. Dynamic parameters that assess volume reactivity, like stroke volume variation (SVV) and pulse pressure variation (PPV), are also increasingly used to monitor volume therapy [6-8]. The aim of this study was to examine the suitability of the established parameters CVP and PCWP and the newer parameters GEDVI, PPV, and SVV to predict changes in cardiac index (CI) and stroke volume index (SVI) after sternotomy in cardiac surgery patients.

## Materials and methods

### Patients

After approval from the ethics committee and patients' written informed consent were obtained, 40 patients were recruited for this study. In these patients, we measured volumetric and dynamic volume parameters.

### Anesthetic procedure

Flunitrazepam (0.5 to 2 mg) was given at night and midazolam (0.1 mg/kg body weight) before surgery as oral premedication. Before induction of general anesthesia, the femoral artery was punctured under local anesthesia for continuous invasive blood pressure measurement. Standardized anesthesia was induced with midazolam (0.05 to 0.1 mg/kg), fentanyl (5 μg/kg), etomidate (0.2 mg/kg), and pancuronium (0.1 mg/kg). Isoflurane (0.6 to 1 volume percentage end tidal) and continuous fentanyl were given to maintain anesthesia. After endotracheal intubation, the patients were ventilated to an end tidal CO_2 _of 35 to 40 mm Hg with a constant tidal volume. A five-channel electrocardiogram and oxygen saturation were continuously recorded. A 4-lumen central venous catheter and a pulmonary artery catheter were inserted via puncture of the internal jugular vein. In all patients included in the study, isovolemic hemodilution using 6% hydroxyaethyl starch (HAES) solution (Voluven^®^; Fresenius Kabi AG, Bad Homburg, Germany) and balancing the preoperative fluid deficit according to clinical criteria with crystalloid volume were performed after induction of anesthesia. We performed hemodilution to achieve a hematocrit below 25% in all patients during cardiopulmonary bypass (CPB) as a local standard. Autologous blood was retransfused after weaning from CPB.

### Determination of cardiac index, global end-diastolic volume, pulse pressure variation, and stroke volume variation

After injection of a cold saline solution, the thermal indicator dilution curve was recorded with a thermistor-tipped catheter in the descending aorta. The CI was determined by a standard thermodilution technique. The calculation of intrathoracic volumes was performed by an analysis of the transit times of the indicator derived from the dilution curve that is recorded in the descending aorta.

Mean transit time (MTt) and exponential downslope time (DSt) of the thermal indicator were recorded. By multiplying CI with the MTt of the indicator, the volume between the sites of injection and indicator detection can be calculated. The intrathoracic thermal volume is based on the thermal indicator curve. Multiplying the CI by the DSt of the thermodilution curve results in the pulmonary thermal volume, which is the largest single mixing volume. GEDV is obtained by subtracting the pulmonary thermal volume from the intrathoracic thermal volume.

Changes in arterial blood pressure induced by mechanical ventilation allow assessment of cardiac preload. In this study, SVV, which is the percentage change between the maximal and minimal stroke volumes (SVs) divided by the average of the minimum and maximum over a floating period of 30 seconds, was recorded. PPV, which was calculated as the difference between the systolic blood pressure and the diastolic blood pressure of the previous beat, was assessed accordingly. SVI was calculated from cardiac output, measured by transpulmonary thermodilution divided by heart rate and body surface area.

### Study design and monitoring

Hemodynamic and volumetric parameters were determined in the study: CVP, PCWP, heart rate, and mean arterial blood pressure were documented immediately after induction of general anesthesia. Moreover, transpulmonary thermodilution of the CI, GEDVI, PPV, and SVV were measured using a commercially available monitor (PiCCO Plus; PULSION Medical Systems AG, Munich, Germany) at this time point. All measurements were repeated 15 minutes after sternotomy. To examine the correlation between changes in hemodynamic and volumetric parameters and CI, differences of each parameter were calculated between the first and second measuring points and correlated to the changes in CI. To assess the predictive capacity of the measured parameters, the absolute values of CVP, PCWP, GEDVI, SVV, and PPV after induction of anesthesia and the relative changes between the two measurements in this study were analyzed with receiver operating characteristic curves. A positive response to the volume load after chest opening was defined as an increase in SVI of 15%, as published previously [9].

### Statistical analysis

All data were given as mean and standard deviation assuming normal distribution, which was verified with the Kolmogorov-Smirnov test. The correlation between changes in hemodynamic and volumetric parameters was analyzed using Pearson's correlation analysis. Changes in individual parameters over the course of the study were determined with the paired *t *test. The predictive capacity of the tested parameters was tested by receiver operating characteristic analysis [9]. The statistical program SPSS (Version 14.0; SPSS Inc., Chicago, IL, USA) was used for the statistical evaluation of all parameters.

## Results

Volumetric and hemodynamic parameters were determined in 40 patients. The first set of measurements was performed directly after inducing anesthesia. Then a mean of 1,364 mL (713 mL) of autologous blood was taken from the patients, and 1,459 mL (605 mL) of HAES 6% and 1,311 mL (742 mL) of crystalloid fluid were substituted as a local standard to account for preoperative deficits and guarantee a hematocrit below 0.25 during CPB. After hemodilution and fluid therapy were concluded, the second set of measurements was performed 15 minutes after sternotomy. Patients' basic characteristics are presented in Table 1.

**Table 1:** Basic characteristics

	Mean	Standard deviation
Age, years	63	10
Male/female gender	36/4	
Height, meters	1.75	0.06
Weight, kg	90	15
Body mass index, kg/m^2^	29.4	4.5
Body surface, m^2^	2.08	0.19

During the study period, there was a significant increase in heart rate and a significant decrease in CVP (Table 2). We also observed a significant decrease in systemic and pulmonary vascular resistances with a concomitant significant increase in CI and SVI (Table 2). GEDVI significantly increased and SVV and PPV significantly decreased after fluid loading (Table 2).

**Table 2:** Hemodynamic measurements

	Induction of anesthesia		After sternotomy		
	Mean	SD	Mean	SD	*P *value

Heart rate, L/minute	66	12	70	17	0.02
Mean arterial blood pressure, mm Hg	72	12	73	14	0.61
Mean pulmonary artery blood pressure, mm Hg	22	4	22	8	0.98
Central venous pressure, mm Hg	12	4	9	4	<0.01
Pulmonary capillary wedge pressure, mm Hg	13	4	12	4	0.19
Cardiac index, L/minute per m^2^	2.16	0.34	3.06	0.76	<0.01
Stroke volume index, mL/m^2^	33.4	6.3	44.2	9.4	<0.01
Systemic vascular resistance, dyn/second per cm^-5^	1,126	240	877	312	<0.01
Pulmonary vascular resistance, dyn/second per cm^-5^	156	72	118	57	<0.01
Global end-diastolic volume index, mL/m^2^	615	99	648	106	0.04
Stroke volume variation, percentage	16	8	10	5	<0.01
Pulse pressure variation, percentage	15	7	9	5	<0.01

SD, standard deviation.

**Figure 1:**
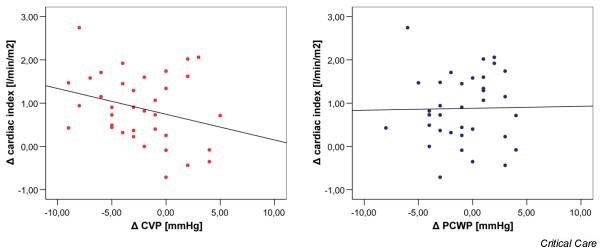
Correlation between changes in the cardiac index, central venous pressure (CVP), and pulmonary capillary wedge pressure (PCWP).

**Figure 2:**
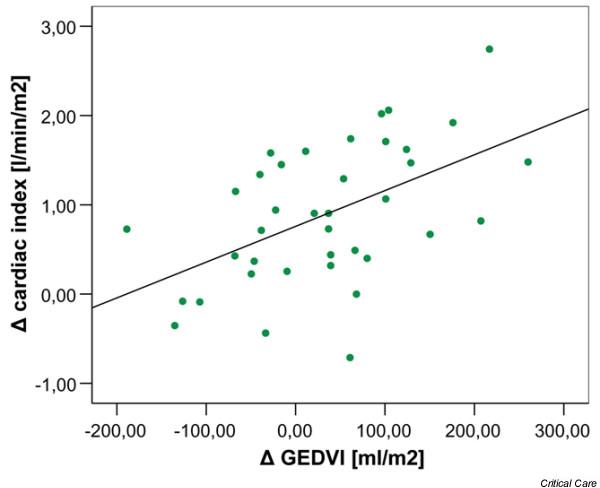
Correlation between changes in the cardiac index and global end-diastolic volume index (GEDVI).

**Figure 3:**
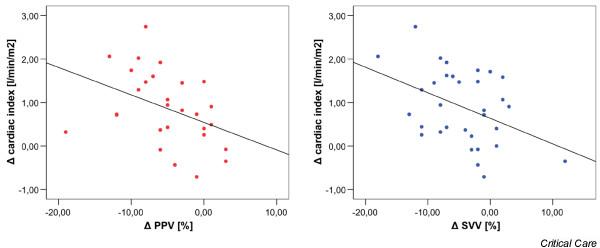
Correlation between changes in the cardiac index, stroke volume variation (SVV), and pulse pressure variation (PPV).

The changes in the CI between the two measuring points did not correlate with changes in the CVP or PCWP (Figure 1). The correlation coefficients were *r *= -0.280 for CVP and *r *= 0.017 for PCWP. In contrast, the increase in GEDVI correlated with the increase in CI (Figure 2). The correlation coefficient here was *r *= 0.518 (*p *< 0.01). Moreover, the decrease in SVV and PPV correlated with an increase in CI with correlation coefficients of *r *= -0.399 (*p *= 0.03) for SVV and *r *= -0.411 (*p *= 0.02) for PPV (Figure 3).

The best prediction of changes in SVI was observed from relative changes in SVV and PPV. Also, the change in GEDVI was predictive for an increase in SVI after fluid loading and sternotomy. None of the absolute parameters after induction of anesthesia was able to predict an increase in SVI after volume loading and chest opening (Table 3).

**Table 3:** Prediction of change in stroke volume index with receiver operating characteristic analysis

	Area under the curve	*P *value	95% confidence interval
CVP induction of anesthesia	0.54	0.69	0.32–0.76
PCWP induction of anesthesia	0.54	0.72	0.31–0.77
GEDVI induction of anesthesia	0.60	0.34	0.38–0.82
SVV induction of anesthesia	0.69	0.10	0.47–0.90
PPV induction of anesthesia	0.67	0.14	0.43–0.91
			
Delta CVP	0.66	0.13	0.45–0.87
Delta PCWP	0.57	0.55	0.35–0.79
Delta GEDVI	0.76	0.01	0.61–0.91
Delta SVV	0.85	0.01	0.69–0.98
Delta PPV	0.80	0.02	0.63–0.96

CVP, central venous pressure; GEDVI, global end-diastolic volume index; PCWP, pulmonary capillary wedge pressure; PPV, pulse pressure variation; SVV, stroke volume variation.

## Discussion

The most important results of this study are that the increase of CI observed after sternotomy and volume loading in our study in cardiac surgical patients did not correlate with changes in CVP and PCWP. In contrast, the changes in GEDVI, SVV, and PPV showed a significant correlation. This is of major clinical interest since the vast majority of anesthesiologists still use filling pressures as a standard monitoring tool for volume management, but as demonstrated by our results, filling pressures might not be the monitoring parameters of choice under open-chest conditions [4]. Only relative changes of SVV, PPV, and GEDVI were able to predict an increase in SVI after sterotomy and volume loading.

Opening the chest causes a decrease in airway pressures and hence in the effects of the mechanical ventilation on venous return. Therefore, after sternotomy, we assumed that PPV and SVV might decrease and CI and SVI might increase alone due to the change in airway pressure. These effects might be regarded as a relative increase in 'cardiac preload' due to a facilitated venous return. This is in line with our findings and was shown previously in an experimental design [10]. Fluid loading is decisively important in cardiac surgical patients to optimize cardiac output and tissue oxygenation [11,12]. With optimal preload, cardiac contractility increases and cardiac work is economized. However, determining left ventricular preload in the clinical routine is particularly difficult during surgery. Filling pressures like CVP and PCWP are normally used as parameters of right and left heart preload. The lack of a correlation of filling pressures as a volume parameter in our study has also been described in other studies and has several reasons: CVP and PCWP depend not only on intravascular volume and peripheral vessel tone but also on right/left ventricular compliance, pulmonary vessel resistance, and intrathoracic pressure. Sakai and colleagues [13] described a significant correlation between increasing PEEP and rising CVP (*r *= 0.88). Furthermore, PCWP depends on the function of the mitral valve and the contractility of the left ventricle. The therapeutic application of vasodilators and vasopressors has been shown to influence measurements [14].

Other studies evaluating volumetric parameters in different surgical areas generally agree with our results. Thus, it was shown that ITBVI and GEDVI correlate better with cardiac preload than filling pressures [15-18]. Moreover, no significant correlation was found between the percentage of changes in CI and the SVI, CVP, and PCWP changes over a 24-hour period in patients after uncomplicated coronary artery bypass surgery [19]. Lichtwarck-Aschoff and colleagues [18] showed for the first time that volumetric parameters like ITBV were superior to filling pressures (CVP and PCWP) for assessing the preload. This was also confirmed in other studies with different patient populations [20-22]. In burn patients, optimized volume and fluid loading are of particular clinical interest since greater volume shifts are seen in these patients over a short time and empirical volume therapy frequently underestimates the real fluid requirement. In these patients, significant correlations were found between ITBVI, CI, and oxygen supply parameters [23], whereas CVP and PCWP failed to predict changes in CI dependent on volume loading. Furthermore, Reuter and colleagues [7] showed that there was a significant decrease in CI and GEDVI after taking autologous blood for hemodilution following sternotomy. Particularly with mechanical ventilation and changes in the intrathoracic pressure, volumetric parameters like ITBVI and GEDVI are clearly superior to filling pressures (CVP and PCWP) for estimating the cardiac preload [24]. This is especially relevant for cardiac surgery patients since intrathoracic pressure markedly changes after sternotomy, which may decisively affect the usability of changes in the filling pressures.

Positive intrathoracic pressure following mechanical ventilation induces a reduction in biventricular preload. This is reflected by variations in the SV. These variations during a defined interval have proven to be useful parameters of cardiac preload [25]. SVV and PPV are parameters derived from changes in SV dependent on mechanical ventilation and have been found useful for monitoring volume reactivity volume therapy in the perioperative phase of cardiac patients [26]. The decrease in SVV and PPV with a concomitant increase in CI following volume loading observed in our study agrees with previous studies. Wiesenack and colleagues [27,28] described a good correlation between changes in SVI and SVV base values after volume loading. In systemic vascular resistance changes, however, the measurement algorithm required recalibration since an increase in the SVI and CI cannot always be reliably determined by pulse contour analysis [27-29]. Furthermore, a significant correlation between SVV and tidal volumes was described before and after fluid loading in a controlled study [30]. In regard to this, tidal volumes were kept constant throughout the study period. Thus, it seems important to observe absolute values and relative changes of SVV and PPV during fluid loading to assess volume loading and to exclude volume overload. Only a decrease in SVV and PPV with a concomitant increase in SVI or CI indicates a volume deficit requiring therapy. This is in line with our finding that only relative changes of PPV, SVV, and GEDVI were predictive of volume responsiveness in our study.

### Limitations

Our study was designed and performed to estimate the effect of a clinical approach of volume loading after isovolemic hemodilution and sternotomy on parameters generally accepted to assess the volume status in cardiac surgical patients. However, this implies that, during our study, two major changes occurred: a clinically assessed volume loading and the sternotomy. Each of these events can have separate hemodynamic effects. Furthermore, we did not measure post-sternotomy catecholamine discharge or a possible decrease in chest wall compliance to explain changes in CI or SVV/PPV. Therefore, no mechanistic insight can be gained from our study. This, however, was not the aim of this clinical study and could be better evaluated by an experimental design. Our study, however, proved that after sternotomy, filling pressures are absolutely not useful for guiding volume therapy and that changes in volumetric or dynamic volume parameters should be used instead.

Thus, our results suggest that CVP and PCWP are not reliable parameters for assessing volume responsiveness in cardiac surgery patients under open-chest conditions. This seems to be especially affected by changes in intrathoracic pressure differences following sternotomy. Changes in volumetric parameters like GEDVI, SVV, and PPV appear to be more reliable for assessing volume responsiveness under these conditions.

## Conclusion

In summary, our results suggest that filling pressures CVP and PCWP are not suitable parameters for monitoring volume therapy during surgery in cardiac patients. Direct volume measurement, such as GEDVI determination or the dynamic volume-reactive parameters SVV and PPV, is clearly superior to the pressure parameters CVP and PCWP, especially under pressure conditions in the thorax changed by sternotomy. Only relative changes in SVV, PPV, and GEDVI were predictive for volume response in this study.

## Key messages

• We observed no correlation between the change of cardiac index (CI) and the changes in the central venous pressure or pulmonary capillary wedge pressure before and after sternotomy and volume loading in patients undergoing coronary artery bypass graft surgery.

• In contrast to this, our study could establish a good correlation between the increase in global end-diastolic volume index (GEDVI) and the increase in CI prior to and after sternotomy and volume loading.

• Moreover, the decrease in stroke volume variation (SVV) and pulse pressure variation (PPV) correlated with an increase in CI.

• Only relative changes in GEDVI, SVV, and PPV predicted a positive volume response.

## Abbreviations

CI = cardiac index; CPB = cardiopulmonary bypass; CVP = central venous pressure; DSt = downslope time; GEDVI = global end-diastolic volume index; HAES = hydroxyaethyl starch; ITBVI = intrathoracic blood volume index; MTt = mean transit time; PCWP = pulmonary capillary wedge pressure; PPV = pulse pressure variation; SV = stroke volume; SVI = stroke volume index; SVV = stroke volume variation.

## Competing interests

The authors declare that they have no competing interests.

## Authors' contributions

MS and CvH prepared the manuscript, carried out the measurements, conceived the study, and performed the statistical analysis. KB, AF, MC, and MK helped with the recruitment of the patients and the drafting of the manuscript. HG participated in the study design and helped with the recruitment of patients. CDS drafted the manuscript and helped with the study design and coordination. All authors read and approved the final manuscript.
